# Amplitude of Intracranial Induced Electric Fields Does Not Linearly Decrease with Age: A Computational Study of Anatomical Effects in Adults

**DOI:** 10.3390/bios15030185

**Published:** 2025-03-13

**Authors:** Jianxu Zhang, Zilong Yan, Anshun Kang, Jian Ouyang, Lihua Ma, Xinyue Wang, Jinglong Wu, Dingjie Suo, Shintaro Funahashi, Wei Meng, Li Wang, Jian Zhang

**Affiliations:** 1 School of Mechatronical Engineering, Beijing Institute of Technology, Beijing 100081, China; jianxuzhang@126.com (J.Z.); yanzilong@bit.edu.cn (Z.Y.); malihua1018@163.com (L.M.); 2 School of Medical Technology, Beijing Institute of Technology, Beijing 100081, China; 3120225707@bit.edu.cn (A.K.); 3120225709@bit.edu.cn (J.O.); 3120221425@bit.edu.cn (X.W.); wujl@bit.edu.cn (J.W.); suo@bit.edu.cn (D.S.); 3 Advanced Research Institute of Multidisciplinary Science, Beijing Institute of Technology, Beijing 100081, China; funahashi@bit.edu.cn; 4 Radiology Department, Harbin Medical University, Harbin Medical University Cancer Hospital, 150 Haping Road, Harbin 150081, China

**Keywords:** transcranial electrical stimulation (tES), age, anatomic parameters, individual variability, electric field

## Abstract

Transcranial electrical stimulation, as a means of neural modulation, is increasingly favored by researchers. The distribution and magnitude of the electric field generated within the brain may directly affect the results of neural modulation. Therefore, it is important to clarify the change trend of the cortical electric field and the determinants of the induced electric field in the endodermis at different ages during the adult life cycle. In this study, we used SimNIBS software to perform MR image segmentation and realistic head model reconstruction on 476 individuals (aged 18 to 88 years old) and calculated the cortical electric field of four electrode montages commonly used in cognitive tasks. We divided all participants into groups by age with a span of 10 years for each group and compared the electric field distribution patterns, electric field intensities, and focalities of the cortexes and regions of interest related to cognitive tasks within groups. The degree of influence of global and local anatomical parameters on the electric field was analyzed using a stepwise regression model. The results showed that, in the cortexes and regions of interest, the variability of electric field distribution patterns was highest in adolescents (<20 years old) and elderly individuals (>80 years old). Moreover, throughout the adult lifespan, the electric field induced by transcranial electrical stimulation did not decrease linearly with age but rather presented a U-shaped pattern. In terms of the entire adult life cycle, compared with global anatomical parameters (intracranial brain tissue volume), local anatomical parameters (such as scalp or skull thickness below the electrode) have a greater impact on the amplitude of the intracranial electric field. Our research results indicated that it is necessary to consider the effects caused by different brain tissues when using transcranial electrical stimulation to modulate or treat individuals of different ages.

## 1. Introduction

Non-invasive transcranial electrical stimulation (tES), compared with the potential risks present in invasive or minimally invasive surgery [1,2], has been increasingly used to treat human psychiatric disorders and regulate the excitability of the human central nervous system. Moreover, multiple studies have demonstrated that tES has a moderate effect size on depressive symptoms (Hedges’s g = 0.651) and achieves a response rate of 53.4% in chronic insomnia patients [3,4]. During tES, a weak current (1–4 mA) is delivered on one or more pairs of electrodes to induce an electric field in the brain to activate specific intracranial targets [5,6]. tES can be categorized into various types according to the form of current used, such as transcranial direct current stimulation (tDCS), transcranial alternating current stimulation (tACS), transcranial random noise stimulation (tRNS), and the recently emerged temporal interference (TI) stimulation [7,8,9,10]. In recent times, various studies have utilized tES to modulate cognitive tasks in both healthy individuals and those with cognitive impairment, and the outcomes have demonstrated a significant level of heterogeneity [11,12,13]. Studies have indicated that only approximately 50% of research findings can be replicated when utilizing tDCS or tACS to modulate cognitive tasks [14,15,16].

In 2009, Michael Koenigs et al. designed a double-blind crossover study that performed tDCS on 25 healthy subjects. The results showed no significant changes in participants’ emotional states and decision making, which was in stark contrast to the results of Lippold and Redfearn [17,18]. Similarly, Sarah Wiethof et al. performed tDCS on 53 healthy subjects in 2014. Using clustering algorithms, they found that half of the subjects had no significant response to tDCS, while the other half demonstrated a beneficial effect [19]. In 2018, Esmaeilpour and colleagues proposed that there is no consensus on whether the current dose (1–2 mA) should be increased linearly to increase the aftereffect of electrical stimulation [20]. In addition, the recent increase in cathode and anode stimulation has yielded varying outcomes [21,22]. An increasing number of studies have shown that various factors, such as the brain state of the individual and the amplitude of the electric field reaching the stimulation target, can lead to different neurophysiological or behavioral outcomes.

In recent years, many studies have used tES to regulate cognitive tasks while combining finite element methods to calculate intracranial electric fields, suggesting a correlation between intracranial electric fields and cognitive performance [23]. At the same time, many studies have used finite element models to calculate the distribution and variation in intracranial electric fields between individuals. Ideal head models based on concentric spheres of different diameters and individual head models constructed from magnetic resonance images were used to predict the distribution and amplitude of intracranial electric fields. The results indicated that differences in individual brain morphology and shape changes may have an impact on the electric field induced by tES [24,25]. In addition, simulation results for young and elderly people have shown that the effect of cerebrospinal fluid (CSF) volume is relatively small for elderly people compared with that for young people [26]. A study focusing specifically on elderly persons showed that the intensity of the electric field in the cerebral cortex is associated with atrophy of the brain [27]. Studies have shown the effect of sex on intracranial electric fields and have also shown that CSF and gray matter (GM) volume have an effect at all ages [28]. In addition, a study evaluated the changes of cerebellar tDCS with aging based on large sample data, and the results showed that healthy aging of the brain has a significant impact on the cerebellar lobes [29]. These studies provide evidence that the cortical electric field is influenced by anatomical parameters. However, there is currently no research investigating the trends of intracranial electric fields across the entire adult life cycle, particularly regarding the impact of healthy aging on cortical and deep brain electric fields based on large sample data, despite the well-known fact that the human brain structure continues to evolve throughout life [30]. Therefore, it is necessary to finely group large sample data to investigate the trends of changes in the intracranial electric field throughout the entire adult life cycle and the main factors affecting the intracranial electric field in different age groups.

In the current study, we reconstructed realistic head models from T1 and T2 MR images of 476 individuals (aged between 18 and 88 years) using SimNIBS software and performed electric field simulation calculations using custom scripts. To obtain more generalized results, we incorporated four tES montages commonly used for neuromodulation. We then grouped individuals per decade and analyzed the distribution patterns and variability of cortical electric fields between and within groups. In addition, we segmented three regions of interest (ROIs) relevant to the cognitive task and extracted the amplitude of the electric field. Finally, we identified trends in cortical electric fields induced by tES across the entire adult life cycle and used stepwise regression to evaluate the extent to which global and local anatomical parameters influence cortical electric fields.

## 2. Materials and Methods

### 2.1. MRI Data Acquisition

High-resolution T1- and T2-weighted structural MRI scans of subjects were obtained from the Cam-CAN repository (Cam-CAN: Cambridge Centre for Ageing and Neuroscience, available at http://www.mrc-cbu.cam.ac.uk/datasets/camcan/, accessed on 18 December 2020). We selected 476 subjects (ages: 18.75–88.92 years) for this study, including complete T1- and T2-weighted structural MRI and diffusion-weighted imaging (DWI) with b-values and b-vectors. Details of the imaging modalities are presented in Appendix A [31,32]. All participants gave written informed consent, and the Cambridgeshire 2 Research Ethics Committee approved the study. Participants’ exclusion criteria of Cam-CAN and more details are shown in a previous publication [31]. Participants were grouped by age in 10-year intervals. The details are shown in Table 1.

### 2.2. Construction of Volume Conductor Models

**Figure 1:**
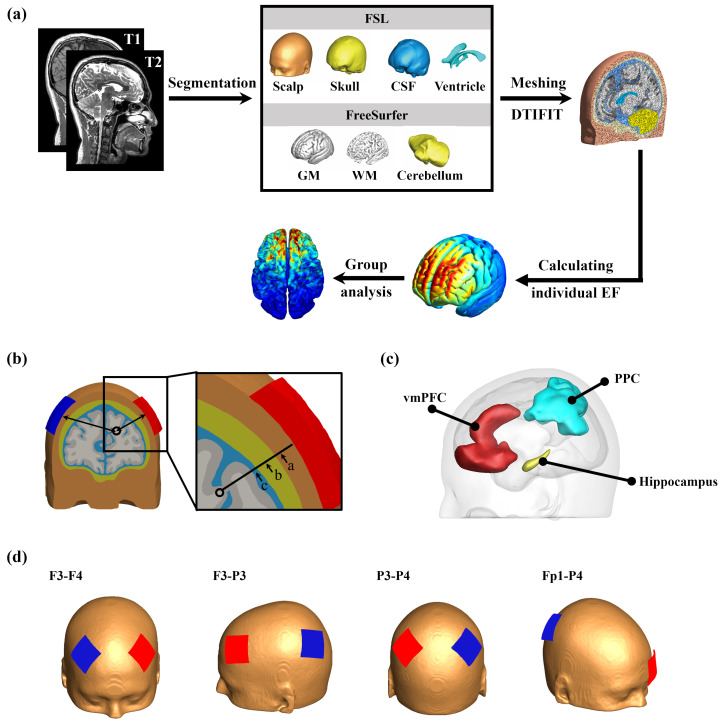
Workflow of analysis. (**a**) Segmentation and calculation process using T1 and T2 magnetic resonance images to segment, assemble, and divide different brain tissues into grids. (**b**) Local anatomical parameters under the electrode, namely, the thickness of the scalp (a), skull (b), and CSF (c). (**c**) Three regions of interest related to cognitive function. (**d**) Four commonly used electrode montages, with red representing the anode and blue representing the cathode.

Head reconstruction was performed using SimNIBS (version 3.2.2, Danish Research Center for Magnetic Resonance, Copenhagen, Denmark) software based on FreeSurfer (version 6.0.0, Martinos Center for Biomedical Imaging, Charlestown, MA, USA) and FSL (version 6.0.5, FMRIB Centre, University of Oxford, Oxford, UK). In this study, realistic finite element head models were reconstructed for 476 subjects. T1- and T2-weighted images were segmented into scalp, skull, CSF, GM, white matter (WM), cerebellum, and ventricles using SimNIBS [33]. In brief, the “mri2mesh” script was used for mesh generation. The default settings were maintained, except for minor adjustments to improve mesh quality in regions with complex geometry, as recommended in the SimNIBS documentation. T1-weighted images were processed using FreeSurfer with its default parameters to segment the cerebellum, gray matter (GM), and white matter (WM). The default recon-all pipeline was used without modification to obtain accurate cortical and subcortical segmentation [34,35]. T2-weighted images were then registered to the corresponding T1-weighted images using FSL’s FLIRT algorithm with default settings. Subsequently, FSL’s BET and BETSURF tools were applied to segment the scalp, skull, cerebrospinal fluid (CSF), and ventricles [36]. Diffusion-weighted images were processed using FSL. The b0 image was extracted following standard procedures, and eddy-current correction and motion correction were applied using FSL’s eddy tool with default parameters [37]. The diffusion tensor was reconstructed from the corrected diffusion data using FSL’s dtifit. The resulting tensor maps (e.g., fractional anisotropy and mean diffusivity) were then registered to the subject’s T1-weighted image using SimNIBS’s built-in registration script. To account for the anisotropic conductivity of WM, the reconstructed diffusion tensor was used as input for SimNIBS, which integrates this information into the finite element model (see Figure 1a for details).

### 2.3. Electrical Field (EF) Computations

Electric field calculations were performed using MATLAB (version R2019b, The MathWorks, Inc., Natick, MA, USA) and customized scripts based on the finite element method and individualized tetrahedral head meshes generated from the structural T1- and T2-weighted images of the subject. First, based on the 10–10 system, four electrode montages commonly used in the study of tES, F3-F4, F3-P3, P3-P4, and Fp1-P4 were used as stimulation electrodes (see Figure 1d for details). These montages were selected because they are frequently employed in cognitive neuromodulation studies, particularly for targeting prefrontal, parietal, and frontoparietal networks, which are critical for working memory, attention, and executive functions. Previous studies have demonstrated their effectiveness in modulating cognitive performance and neural activity in similar experimental paradigms [38,39,40]. The size of the electrode was set to a 5 × 5 cm pad (σ = 29.4 S/m) with a thickness of 2 mm, placed on the scalp with a 5 mm saline gel interface (σ = 1 S/m). The stimulation current was adjusted to ±2 mA. Although it has been shown that there is a positive correlation between changes in WM conductivity and aging [41], we have mainly focused on evaluating the effects of age-related changes in anatomy on the electric field. Consistent with prior simulation studies [42], we adopted fixed conductivity values: σ(scalp) = 0.465 S/m, σ(skull) = 0.01 S/m, σ(cerebrospinal fluid) = 1.654 S/m, σ(GM) = 0.275 S/m, and σ(WM) = 0.126 S/m.

### 2.4. Anatomical Parameters

After completing the segmentation of T1 and T2 MR images, we defined global and local anatomical parameters. The volumes of GM, WM, cerebellum, and ventricles obtained by segmentation were divided by the total intracranial volume (*TIV*), and the ratio obtained was used as the global anatomical parameter (Equation (1)). We defined the thickness of the tissue under the cathode and anode, including the thickness of the scalp, skull, and cerebrospinal fluid under the anode and cathode, as local anatomical parameters (Figure 1b).

(1) Relativetissuevolume=(tissuevolume)/TIV

### 2.5. ROI Definition

In the present study, we included the ventromedial prefrontal cortex (vmPFC, Brodmann: 11 24 25 32 33), posterior parietal cortex (PPC, Brodmann: 1 2 3 5 7 39 40), and left hippocampus as ROIs because studies have demonstrated their effects on cognitive function [43]. The Brodmann atlas was registered to the Desikan–Killiany atlas using FreeSurfer, and the registered ROI was used to extract the amplitudes of the individual electric fields (Figure 1c).

### 2.6. Statistical Analysis

Considering the small size of the ROI, which is sensitive to electric field artifacts, the peak electric field was defined as the 99th percentile value of the simulated electric field distribution. This threshold was chosen to provide a robust and representative measure of peak stimulation intensity while minimizing the influence of extreme outliers or localized numerical artifacts that can occur during the simulation process. Unlike using the absolute maximum value—which may be overly sensitive to singular anomalies—the 99th percentile offers a stable estimate that better reflects the true peak of the electric field [26,44,45]. In addition, we define focality as the volume of the GM region with electric field amplitude higher than the 75th percentile [26]. We use the intersubject registration method to map the calculated cortical electric field of each individual onto the surface of the fs average to obtain the distribution pattern of the cortical electric field. To compare the electric fields of different age groups, the trend of the electric field with age was investigated by calculating the mean of the electric field amplitude. Finally, for each montage, we constructed an individual stepwise linear regression model using MATLAB’s ‘stepwiselm’ function to examine the relationship between the cortical electric field amplitude and all anatomical parameters. Our approach utilized a backward elimination procedure, where variables with a *p*-value greater than 0.05 were sequentially removed from the model. At each step, coefficients and *p*-values were recalculated until all remaining predictors met the significance criterion (*p* < 0.05). Additionally, the ‘stepwiselm’ function automatically excluded any predictors that were linearly dependent on others in the model, regardless of their individual *p*-values. The correlation coefficients in the results indicate the strength and direction of the linear relationships between selected predictors and the dependent variable. Positive values suggest a direct relationship, whereas negative values indicate an inverse relationship.

All statistical analyses were performed using MATLAB (version 2019b). Independent samples *t*-tests were conducted to compare differences in electric field variability across age groups and electrode montages. Statistical significance was set at *p* < 0.05.

## 3. Results

### 3.1. Cortex Electric Field Distribution

**Figure 2:**
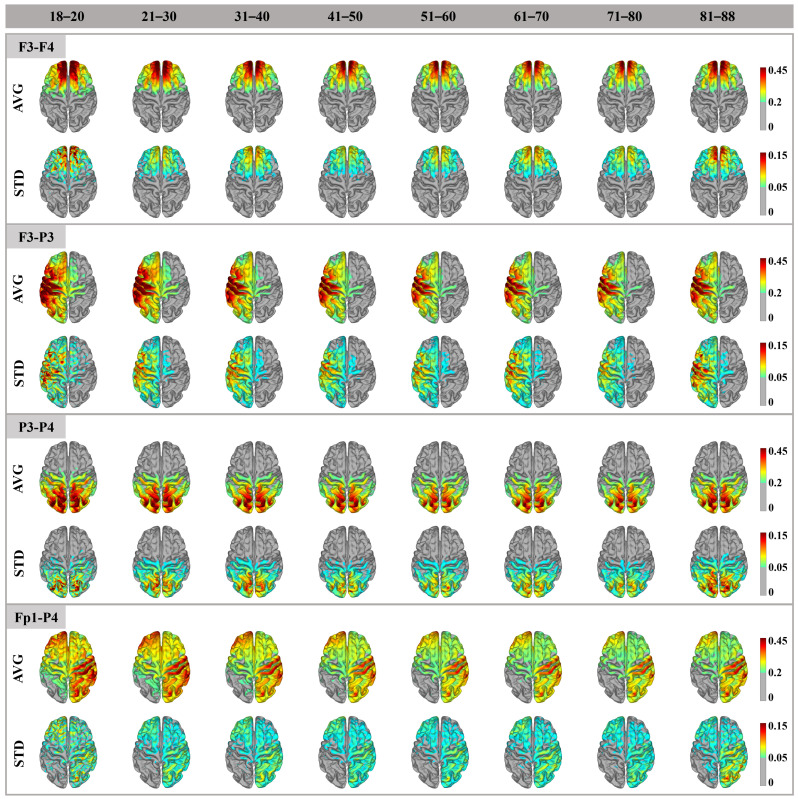
Electric field distribution patterns in different age groups. The average (AVG, 0.2–0.45 V/m) and standard deviation (STD, 0.05–0.15) of cortical electric field amplitudes obtained by age groupings of four montages. The electric field value was extracted from individuals using the 99th percentile electric field value as the peak to eliminate outliers in the electric field.

Figure 2 shows a qualitative analysis of the distribution and variation in cortical electric fields induced by four electrode montages in different age groups. The electric field amplitude of each participant was plotted on a standard fs-average template to obtain the average electric field for each age group. For all electrode montages, (1) the electric field distribution patterns between age groups were very similar, with high electric field amplitudes distributed below and around the two electrodes; (2) the amplitude and focality of the electric field showed the same trend of change, often accompanied by larger amplitude and focality in the younger group. However, the amplitude of the electric field does not decrease linearly with age. As expected, the maximum cortical electric field amplitude varies with electrode position, but the maximum electric field amplitude is concentrated between the two electrodes. Although the Fp1-P4 electrode also showed a high electric field amplitude at the bottom, its maximum value was also in the path between the two electrodes. It was obvious that the position of the electrode configuration affected the focality size of the electric field in the cortex. The smaller the distance between the anode and cathode was, the more focused the electric field was. As expected, variation in the electric field between individuals occurred in areas with higher cortical electric field amplitudes, especially in the frontal and parietal regions. This suggested that, in some subjects, there is a high electric field, but in others, the position and/or direction of the electric field is variable. We noticed that there was generally greater variability in the youngest cohort (18–20 years) and the oldest cohort (81–88 years), which may be due to the greater variability of individuals in these two age groups. For the Fp1-P4 electrode montages, due to their wide electric field distribution, they also had a wide range of variation regions.

**Figure 3:**
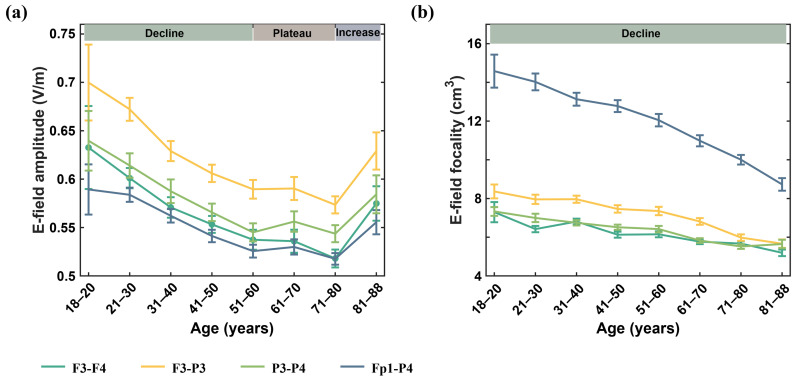
Variation trend of cortical electric field amplitude (**a**) and focality (**b**) with age in four electrode configurations.

Figure 3 and Table 2 display the trends in changes for both the maximum amplitude and focality of the cortical electric field. Specifically, Figure 3a demonstrates the trend for the maximum amplitude of the electric field. The amplitude of the cortical electric field can be categorized into three different periods based on age. First, before the age of 60, there was an approximately linear decreasing trend of the peak value of the electric field with age. Second, between the ages of 61 and 80, the decline rate of the peak electric field slowed and reached its lowest value at the ages of 71–80. Last, after the age of 81, there was an approximately linear upward trend of the peak value of the electric field. We discovered that the positioning of the electrodes, whether on the ipsilateral or contralateral side of the brain, did not affect the trend. Nevertheless, the intracranial electric field induced by the ipsilateral electrode (F3-P3) was significantly higher than that of the contralateral electrode (F3-F4, P3-P4, Fp1-P4) across all age groups (all *p* < 0.05). Importantly, the electric field induced by the parietal electrode (P3-P4) was significantly higher than that of the frontal parietal electrode (Fp1-P4) in all age groups (all *p* < 0.05), likely due to the proximity of the parietal electrodes. Figure 3b displays the trend of changes in the electric field focality, which aligns with previous research indicating an increasingly focused trend as age increases. As anticipated, the frontoparietal electrode with greater electrode spacing and the ipsilateral electrode produced a larger focality, in line with typical tES. We conducted further analysis of sex differences in the electric field and found no differences in amplitude or focality of the electric field for the F3-F4 electrode. However, sex differences in electric field values and focality existed for other electrode montages, particularly in certain age groups. Compared with electric field amplitude, sex differences in focality occurred in more age groups (refer to Appendix A Table A1 for details).

### 3.2. ROI Electric Field Amplitude

**Figure 4:**
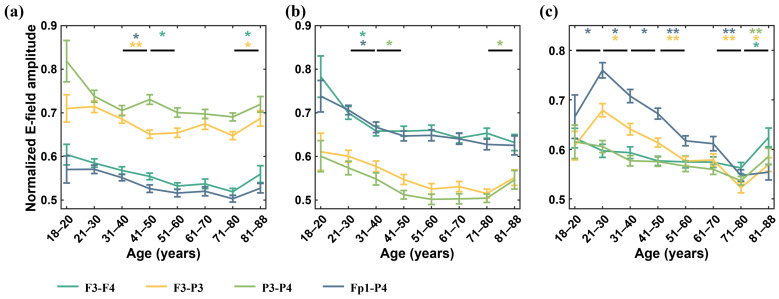
Variation trend of electric field amplitude in ROIs is shown in the following regions: (**a**) ventromedial prefrontal cortex (vmPFC), (**b**) posterior parietal cortex (PPC), and (**c**) hippocampus. (* *p* < 0.05, ** *p* < 0.01).

We selected the ROI in the left hemisphere uniformly as our analysis target and normalized the electric field amplitude to better observe the trends of changes. Figure 4 depicts the trend of electric field amplitude variation at three different ROIs. As envisioned, when the ROI was located below or between the electrodes, a substantial induced electric field was obtained at the ROI (see Table 3 for details). Similar to changes in cortical electric field trends, the electric field value at the ROI did not decrease linearly throughout the entire age cycle. For both the vmPFC and PPC, the electric field value at the ROI exhibited an approximately linear decreasing trend before 60 years of age. At ages 61–80, the decrease rate of the electric field peak slowed and hit its lowest value between ages 71 and 80. After age 81, the electric field peak presented an approximately linear upward trend. At the same time, it was observed that, for the vmPFC, there was a slight increase in average amplitude at ages 61–70 years compared with that at ages 51–60, but it was not statistically significant (*p* = 0.217). The P3-P4 electrode exhibited a slight increase in average amplitude at ages 41–50 compared with that at ages 31–40, but it was not statistically significant (*p* = 0.114). For the hippocampus located deep in the brain, the mean electric field amplitudes of F3-P3 (*p* = 0.057) and Fp1-P4 (*p* = 0.038) were smaller at the ages of 18–20 compared with those at the ages of 21–30.

### 3.3. Anatomical Parameters

**Figure 5:**
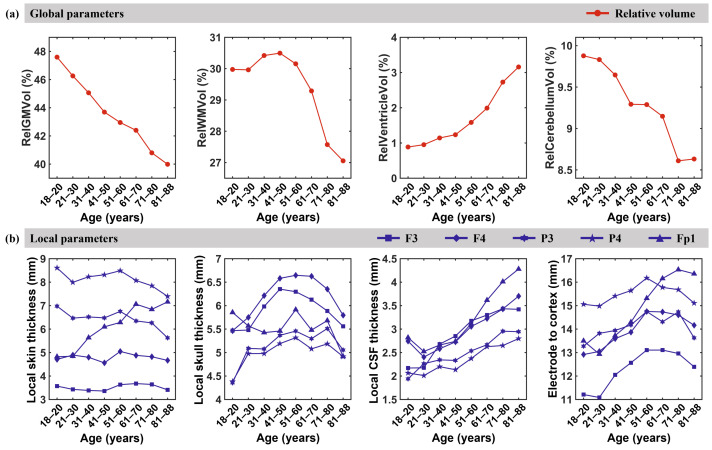
Anatomic parameters: (**a**) global anatomical parameters; (**b**) local anatomical parameters.

We employed FreeSurfer software and a custom MATLAB code to extract anatomical parameters of intracranial brain tissues and nonbrain tissues. This included various global anatomical parameters, such as relative GM volume, relative WM volume, relative cerebellum volume, and relative ventricle volume, in addition to local anatomical parameters, such as local scalp thickness, local skull thickness, local CSF thickness, and electrode-to-cortex distance. We discovered that GM demonstrated rapid decline throughout the entire lifespan until age 50. At ages 51–70, the rate of relative GM volume reduction decreased, but after age 70, relative GM volume further declined due to aging-related brain atrophy. For relative WM volume, an approximate inverted U-shaped model was observed throughout the entire lifespan, exhibiting a rising trend before age 50. After 50 years of age, there was a significant decline in relative WM volume due to brain atrophy caused by aging. For relative ventricle volume, there was an upward trend throughout life, particularly in those older than 60, where the rate of increase rose. This may be attributed to the hastened degeneration of GM and WM after the age of 60. The relative cerebellar volume also exhibited a decreasing trend throughout the entire lifespan, similar to the relative GM volume. Prior to the age of 50, the relative cerebellum volume rapidly decreased and reached a plateau between the ages of 51 and 70, followed by a decline between the ages of 71 and 88 (see Figure 5a). Figure 5b demonstrates the age-related changes in local anatomical parameters. The anatomical parameters under electrodes F3, F4, P3, and P4 exhibited similar trends. Local scalp thickness displayed slight fluctuations before the age of 60, with no significant difference between adjacent age groups. However, after the age of 60, local scalp thickness gradually decreased. The thickness of the local skull exhibited an inverted U-shaped pattern whereby it increased before the age of 60, reaching its maximum at approximately 60. Additionally, the thickness of CSF below all electrodes increased after the age of 20, notwithstanding the noticeable decline in CSF thickness below electrodes F4 and Fp1 between ages 21 and 30 in comparison with that at the ages of 18–20. Notably, the anatomical measurements under electrode Fp1 located below the prefrontal lobe are dissimilar to those of other electrodes. The scalp thickness under this electrode exhibited an increasing trend until the age of 70 and then stabilized. Additionally, the skull thickness beneath Fp1 fluctuated slightly until the age of 80 and decreased thereafter. It is important to highlight that substantial variations in scalp thickness were detected at different electrodes.

### 3.4. Correlation Between Cortical E-Field Amplitude and Anatomical Parameters

**Figure 6:**
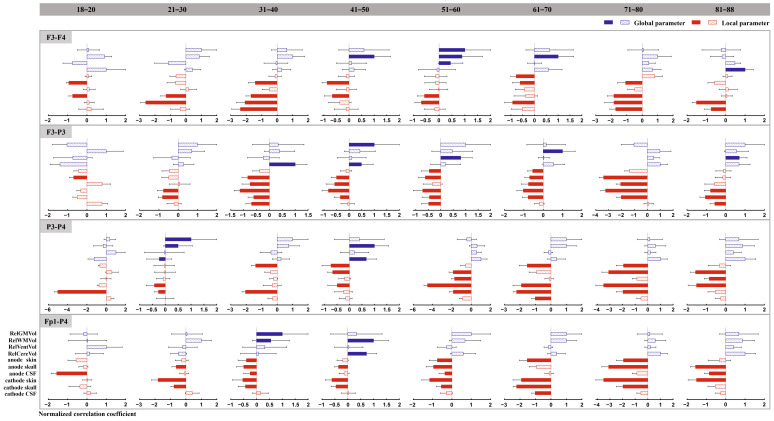
Results of the correlation between anatomical parameters and cortical electric field amplitude. Blue represents global anatomical parameters, while red represents local anatomical parameters. Filled bar indicates that this parameter significantly affects the intracranial induced electric field. Abbreviations: relative GM volume (RelGMVol), relative WM volume (RelWMVol), relative ventricle volume (RelVentVol), relative cerebellum volume (RelCereVol), anode inferior scalp thickness (anode skin), anode inferior skull thickness (anode skull), anode inferior cerebrospinal fluid thickness (anode CSF), cathode inferior scalp thickness (cathode skin), cathode inferior skull thickness (cathode skull), and cathode inferior cerebrospinal fluid thickness (cathode CSF).

For all electrode montages, we used a stepwise linear regression model to assess the correlation between cortical electric field amplitude and anatomical parameters. Figure 6 displays the standardized correlation coefficients between anatomical parameters and cortical electric field amplitudes for various age groups. Our results indicated that (1) local anatomical factors have a greater influence on the amplitude of intracranial electric fields in adults than global anatomical factors. Especially in the age groups of 18–20 and 71–80, the electric field was influenced solely by local anatomical parameters. (2) The amplitude of the electric field was primarily determined by varying factors among different age groups. For instance, in the younger age range (18–20), taking electrode montage F3-F4 as an example, the amplitude of the cortical electric field was primarily influenced by local scalp thickness (cathode) and local skull thickness (anode). However, from ages 51 to 60, both global anatomical parameters (relative GM volume, relative WM volume, and relative ventricle volume) and local anatomical parameters (thickness of scalp and skull) jointly impacted the magnitude of cortical electric fields. Importantly, the primary factors that affect cortical electric fields varied depending on the electrode montage used. In summary, the size of the cortical electric field was influenced by multiple anatomical factors rather than a single factor (see Figure 6 for details).

## 4. Discussion

In this computational study, MR images were utilized from 476 individuals ranging in age from 18 to 88 years old, and they were divided into 10-year age groups. We investigated the changes in cortical electric field intensity and focality induced by a traditional tES electrode montage, as well as the trends in electric field intensity in three ROIs. Using a stepwise linear regression model, we analyzed the main factors influencing the cortical electric field across distinct age groups. Our research mainly revealed the following three findings: (1) the amplitude of the cortical electric field varied among different age groups, (2) the relationship between cortical electric field amplitude and age did not demonstrate a simple linear decrease but presented a U-shaped shape, and (3) the main factors affecting the cortical electric field changed with age groups.

### 4.1. Distribution Pattern and Amplitude of the Electric Field

Compared with previous computational studies that solely utilized T1 magnetic resonance images for segmentation modeling, we incorporated T2 magnetic resonance images in this study to enhance segmentation accuracy and computational precision [46]. Our findings revealed that the distribution patterns induced by all electrode montages of cortical electric fields were remarkably analogous among age groups, aligning with previous research outcomes [26]. However, these studies did not examine variations across different age cohorts [24] or merely categorized age into basic two or three groups [26,28]. Furthermore, upon further refining the classification, we discovered significant individual differences in the youngest (18–20 years old) and oldest (81–88 years old) age groups. These differences may be attributed to variations in brain structure resulting from brain development or atrophy [30]. Additionally, we found that the electric field amplitude did not exhibit a solely linear decrease with age. Instead, it was separated into the following three distinct periods: a declining phase (11–60 years old), a plateau phase (61–80 years old), and a rising phase (81–88 years old). The reason for this pattern was that, until the age of 60, the distance between the electrode and the cortex continued to increase as an individual aged. However, between the ages of 61 and 80, the distance from the electrode to the cortex appeared to stabilize. After the age of 81, however, the distance from the electrode to the cortex began to decrease (see Figure 5b). An additional factor contributing to the increase in electric field amplitude after the age of 81 could be attributed to the rise in CSF, which plays a significant role in the current shunt entering the cortex [47]. The electric field induced by the ipsilateral electrode montage is significantly greater than that of the contralateral electrode. This may be due to the presence of more CSF in the brain’s longitudinal fissure, which shunts most of the current. This is consistent with previous studies indicating that CSF has a notable effect on intracranial electrical fields [24,48]. In addition, we found that, in the three ROIs, the trend of electric field changes was basically consistent with the trend of cortical electric field changes. Only the amplitude of the electric field in the deep brain area of the hippocampus at ages 18–20 years was smaller than that at ages 21–30 years. This may be because the hippocampus is still growing before the age of 20, and then its volume decreases slightly [49]. Overall, our findings indicated that the intracranial electric field induced by tES does not decrease linearly with age, extending previous research findings [26,27,28].

### 4.2. Global and Local Anatomical Parameters

Our findings reveal distinct trajectories of age-related anatomical changes across global and local brain structures, which may reflect both neurodevelopmental and neurodegenerative processes. These patterns provide critical insights into the dynamic interplay between structural decline and functional resilience during aging. In this study, we found that, from the age of 18, the relative GM volume tended to decrease linearly with age, which is basically consistent with the findings of previous studies [30]. The rapid decline in relative gray matter (GM) volume before age 50 may be due to the contraction of large neurons [50,51]. However, the attenuated decline between ages 51 and 70 suggests a potential compensatory mechanism, possibly mediated by neuroplasticity or vascular adaptations. The accelerated GM loss after 70 likely marks the exhaustion of such compensatory reserves, consistent with amyloid-beta accumulation and tauopathy patterns in preclinical Alzheimer’s disease [52,53]. Notably, the inverted U-shaped trajectory of white matter (WM) volume—peaking around age 50—parallels the timeline of myelination cycles and oligodendrocyte dysfunction. The post-50 WM decline may reflect demyelination and axonal degeneration, which have been linked to age-related cognitive slowing [30,54,55,56]. The divergent trajectories of ventricles’ expansion (continuous increase) versus cerebellar atrophy (early decline followed by stabilization) highlight region-specific vulnerabilities. While ventricles’ enlargement is a hallmark of global brain atrophy, the cerebellum’s early volumetric loss may relate to its high metabolic demand and susceptibility to oxidative stress [30,57,58,59]. These findings extend prior cross-sectional studies by delineating non-linear phase transitions in aging trajectories. The local anatomical parameters displayed nonuniformity. Except for the frontal lobe electrode Fp1, there were no significant changes in local scalp thickness before the age of 60. However, a decreasing trend was observed at age 60. The scalp thickness of the frontal lobe demonstrated an approximately linear increasing trend with age. Similarly, relative skull thickness, except frontal lobe electrode Fp1, generally increased and then decreased with age. The peak typically occurred between ages 41 and 60. The thickness of the skull beneath the frontal lobe electrode Fp1 exhibited a complex pattern of initially decreasing, then increasing, and ultimately decreasing again. This finding may be attributed to local pressure changes from the reduction in GM volume and CSF increase [60]. The thickness of local CSF showed an increasing trend with age, which is consistent with previous research results in which the volume of CSF increased with age [61]. We estimated the distance from the electrode to the cortex as a sum of the thickness of the local skin, skull, and CSF. Similarly, the distance from the local electrode to the cortex tends to increase and then decrease due to the greater thickness of the local scalp, which plays a dominant role. However, the approximate trend of the distance from the frontal lobe electrode to the cortex was upward.The electrode-specific variations in scalp/skull thickness and CSF dynamics (Figure 5b) carry critical implications for EEG signal interpretation. The inverted U-shaped skull thickness under most electrodes (peak at 60 years) may attenuate age-related signal attenuation, as thicker skulls reduce conductivity. Conversely, the prefrontal Fp1 montage exhibited unique trends—prolonged scalp thickening until age 70 and delayed skull thinning—which could partially explain reported age-related frontal EEG power shifts [62,63].

### 4.3. Correlation Between Anatomical Parameters and Cortical Electric Field

Previous research has demonstrated a negative correlation between age and electric field amplitude, indicating that older individuals exhibit lower amplitudes [26,28,64]. Aprinda Indahlastari et al. [27] conducted a study on a sample of 587 healthy elderly individuals, wherein the results indicated a negative correlation between electric field values and brain atrophy. These studies provide important preliminary evidence for the factors that determine the electric field based on individual differences. Sagarika Bhattacharjee et al. [28] analyzed electric fields to examine sex differences. In the elderly group, women experienced a larger induced electric field than men, while no sex difference was detected in the middle-aged group. However, these studies only evaluated a small number of participants of the same age or only divided them into young and elderly groups. Moreover, there is no relevant information on global or local anatomical parameters. Our study confirmed these results, namely, the influence of anatomical parameters on the intracranial electric field. Our research results extended the above results by grouping 476 subjects into groups at 10-year intervals and investigated the effects of global and local anatomical parameters on intracranial evoked electric fields. As previously studied, it has been found that relative GM volume, relative WM volume, relative ventricle volume, and relative cerebellum volume affect the amplitude of intracranial electric fields across different age groups. In addition, our results showed that the global anatomical parameter changes caused by brain atrophy are only a partial factor affecting the intracranial induced electric field. In particular, we found that local anatomical parameters have a greater impact on the intracranial induced electric field, with local scalp thickness and skull thickness almost dominating the changes in the intracranial electric field [48,65]. The variation in results suggested that accurate modeling and calculation of the subjects is necessary to achieve similar amplitudes of the electric field across diverse subjects.

### 4.4. Limitations

First, the results of this study are derived from finite element modeling of MRI image data from publicly available datasets. It is still necessary to confirm through relevant cognitive experiments whether these results induce corresponding behavioral and neurophysiological changes. While simulations are valuable for understanding trends in cortical electric field distribution, future studies that incorporate actual physiological data across age groups would provide critical validation of the simulated results and enhance the relevance of our findings to functional outcomes. In addition, due to the specificity of the data, the small sample size of the youngest age group may not be sufficient to detect between-group differences with moderate effect sizes. We recommend that future studies expand the sample size of the youngest group. Second, the electric field amplitude considered in this study refers to the vector norm of the electric field and does not encompass other directions of the electric field. Studies have shown that the structure of specific brain regions (such as gyrus or sulcus) or the configuration of high-definition electrodes may be related to the direction of the electric field. However, when the electrical field covers a wide area of the cortex, the directionality of the electric field is not a significant factor [66]. Finally, another potential limitation of our study is the use of standardized tissue conductivity values rather than age-dependent conductivity measures. While evidence suggests that tissue conductivity might change with aging, the lack of robust, validated models across the adult lifespan constrains our ability to incorporate such variations. However, our primary objective was to evaluate the influence of anatomical differences on electric field distribution, and existing studies have shown that changes in th conductivity of head tissues such as scalp, compact bone skull layer, and brain tissue may be negligible [67]. We believe that, in future research, a more comprehensive understanding of age-related changes in cortical electric fields may benefit from including tissue conductivity as a variable.

## 5. Conclusions

In summary, in the current research based on computational models, we found that (1) the induced electric field varies among different age groups and (2) the intracranial induced electric field amplitude does not decrease linearly with age but rather presents a U-shaped pattern. However, the focality of the induced electric field decreases with age. (3) The main factors affecting intracranial induced electric fields varied among different age groups, and compared with global anatomical parameters, local anatomical parameters had a greater impact on the amplitude of intracranial electric fields. Our results promote an understanding of the individual variability of tES effects in adults and contribute to understanding the individualized differences in intracranial evoked electric fields.

## Figures and Tables

**Table 1:** Age range, mean, and standard deviation (SD) of subjects by age.

Age Range	Number of Subjects	Sex (M/F)	Mean Age (SD)
18–20	7	5/2	19.38 (0.69)
21–30	49	17/32	26.66 (2.55)
31–40	76	41/35	35.96 (2.87)
41–50	79	39/40	46.21 (2.83)
51–60	73	40/33	55.67 (2.95)
61–70	72	36/36	65.78 (2.95)
71–80	84	38/46	76.43 (3.13)
81–88	36	19/17	84.11 (1.99)
Total	476	235/241	54.78 (18.53)

**Table 2:** Means (standard error) [95% CI] of E-field amplitude (V/m) and focality (cm^3^) for different age groups.

Age (Years)	E-Field Amplitude				E-Field Focality			
	F3-F4	F3-P3	P3-P4	Fp1-P4	F3-F4	F3-P3	P3-P4	Fp1-P4
18–20	0.633 (0.043)	0.700 (0.039)	0.640 (0.031)	0.589 (0.026)	7.295 (0.522)	8.365 (0.358)	7.331 (0.228)	14.580 (0.851)
	[0.53, 0.74]	[0.60, 0.80]	[0.53, 0.65]	[0.56, 0.72]	[6.02, 8.57]	[7.49, 9.24]	[12.50, 16.66]	[6.77, 7.89]
21–30	0.601 (0.010)	0.672 (0.012)	0.614 (0.013)	0.584 (0.007)	6.419 (0.163)	7.955 (0.240)	6.997 (0.216)	14.024 (0.434)
	[0.58, 0.62]	[0.65, 0.70]	[0.57, 0.60]	[0.59, 0.64]	[6.09, 6.75]	[7.47, 8.44]	[13.15, 14.90]	[6.56, 7.43]
31–40	0.571 (0.011)	0.629 (0.010)	0.587 (0.012)	0.562 (0.007)	6.808 (0.152)	7.961 (0.185)	6.745 (0.137)	13.129 (0.332)
	[0.55, 0.59]	[0.61, 0.65]	[0.55, 0.58]	[0.56, 0.61]	[6.50, 7.11]	[7.59, 8.33]	[12.47, 13.79]	[6.47, 7.02]
41–50	0.553 (0.009)	0.606 (0.009)	0.566 (0.009)	0.541 (0.006)	6.132 (0.155)	7.460 (0.193)	6.517 (0.132)	12.774 (0.306)
	[0.54, 0.57]	[0.59, 0.62]	[0.53, 0.55]	[0.55, 0.58]	[5.82, 6.44]	[7.08, 7.84]	[12.16, 13.38]	[6.25, 6.78]
51–60	0.537 (0.010)	0.590 (0.010)	0.545 (0.009)	0.526 (0.007)	6.152 (0.154)	7.354 (0.216)	6.419 (0.165)	12.047 (0.321)
	[0.52, 0.56]	[0.57, 0.61]	[0.51, 0.54]	[0.53, 0.56]	[5.85, 6.46]	[6.92, 7.78]	[11.41, 12.69]	[6.09, 6.75]
61–70	0.536 (0.012)	0.590 (0.012)	0.556 (0.011)	0.530 (0.008)	5.770 (0.128)	6.815 (0.174)	5.829 (0.121)	10.983 (0.288)
	[0.51, 0.56]	[0.57, 0.61]	[0.51, 0.55]	[0.54, 0.58]	[5.52, 6.02]	[6.47, 7.16]	[10.41, 11.56]	[5.59, 6.07]
71–80	0.518 (0.009)	0.573 (0.009)	0.544 (0.009)	0.518 (0.006)	5.671 (0.117)	5.980 (0.166)	5.520 (0.121)	10.004 (0.248)
	[0.50, 0.54]	[0.56, 0.59]	[0.51, 0.53]	[0.53, 0.56]	[5.44, 5.90]	[5.65, 6.31]	[9.51, 10.50]	[5.28, 5.76]
81–88	0.575 (0.018)	0.629 (0.019)	0.584 (0.020)	0.556 (0.012)	5.194 (0.170)	5.668 (0.218)	5.649 (0.221)	8.728 (0.326)
	[0.54, 0.61]	[0.59, 0.67]	[0.53, 0.58]	[0.54, 0.62]	[4.85, 5.54]	[5.23, 6.11]	[8.07, 9.39]	[5.20, 6.10]

**Table 3:** Means (standard error) of E-field amplitude (V/m) in ROIs for different age groups.

ROI	Age (Years)	F3-F4	F3-P3	P3-P4	Fp1-P4
vmPFC	18–20	0.348 (0.014)	0.355 (0.016)	0.102 (0.006)	0.523 (0.028)
	21–30	0.337 (0.006)	0.357 (0.007)	0.092 (0.002)	0.523 (0.009)
	31–40	0.327 (0.005)	0.343 (0.005)	0.088 (0.001)	0.505 (0.007)
	41–50	0.319 (0.004)	0.326 (0.005)	0.091 (0.001)	0.482 (0.008)
	51–60	0.307 (0.004)	0.327 (0.005)	0.088 (0.001)	0.473 (0.008)
	61–70	0.310 (0.006)	0.338 (0.006)	0.087 (0.001)	0.477 (0.009)
	71–80	0.299 (0.005)	0.324 (0.005)	0.086 (0.001)	0.461 (0.007)
	81–88	0.322 (0.011)	0.344 (0.009)	0.090 (0.002)	0.483 (0.010)
PPC	18–20	0.199 (0.012)	0.477 (0.033)	0.507 (0.030)	0.327 (0.016)
	21–30	0.178 (0.004)	0.469 (0.011)	0.484 (0.013)	0.313 (0.005)
	31–40	0.167 (0.003)	0.451 (0.010)	0.463 (0.012)	0.295 (0.005)
	41–50	0.167 (0.003)	0.428 (0.009)	0.432 (0.008)	0.286 (0.005)
	51–60	0.168 (0.003)	0.411 (0.010)	0.424 (0.010)	0.287 (0.006)
	61–70	0.163 (0.003)	0.415 (0.009)	0.424 (0.010)	0.283 (0.006)
	71–80	0.166 (0.003)	0.402 (0.008)	0.426 (0.008)	0.278 (0.005)
	81–88	0.161 (0.005)	0.431 (0.014)	0.461 (0.018)	0.277 (0.010)
Hippocampus	18–20	0.177 (0.006)	0.269 (0.013)	0.142 (0.008)	0.335 (0.022)
	21–30	0.169 (0.004)	0.301 (0.006)	0.140 (0.003)	0.382 (0.008)
	31–40	0.168 (0.003)	0.284 (0.005)	0.133 (0.002)	0.356 (0.007)
	41–50	0.164 (0.003)	0.272 (0.004)	0.133 (0.002)	0.338 (0.006)
	51–60	0.163 (0.003)	0.255 (0.004)	0.130 (0.003)	0.310 (0.005)
	61–70	0.163 (0.003)	0.256 (0.006)	0.129 (0.002)	0.307 (0.007)
	71–80	0.159 (0.003)	0.231 (0.005)	0.124 (0.002)	0.275 (0.006)
	81–88	0.177 (0.005)	0.252 (0.006)	0.135 (0.004)	0.278 (0.008)

## Data Availability

High-resolution T1- and T2-weighted structural MRI scans of subjects were obtained from the Cam-CAN repository (Cam-CAN: Cambridge Centre for Ageing and Neuroscience, available at http://www.mrc-cbu.cam.ac.uk/datasets/camcan/, accessed on 18 December 2020).

## Abbreviations

The following abbreviations are used in this manuscript:

tES	transcranial electrical stimulation
tDCS	transcranial direct current stimulation
tACS	transcranial alternating current stimulation
tRNS	transcranial random noise stimulation
TI	temporal interference
CSF	cerebrospinal fluid
GM	gray matter
WM	white matter
ROIs	regions of interest
DWI	diffusion-weighted imaging
TIV	total intracranial volume
vmPFC	ventromedial prefrontal cortex
PPC	posterior parietal cortex
RelGMVol	relative GM volume
RelWMVol	relative WM volume
RelVentVol	relative ventricle volume
RelCereVol	relative cerebellum volume

## Appendix A

T1-weighted structural image: A high-resolution 3D T1-weighted structural image is acquired using a Magnetization Prepared RApid Gradient Echo (MPRAGE) sequence with the following parameters: repetition time (TR) = 2250 ms; echo time (TE) = 2.99 ms; inversion time (TI) = 900 ms; flip angle = 9 degrees; field of view (FOV) = 256 mm × 240 mm × 192 mm; voxel size = 1 mm isotropic; GRAPPA acceleration factor = 2; and acquisition time of 4 min and 32 s. T2-weighted structural image: A high-resolution 3D T2-weighted structural image is acquired with a SPACE sequence with the following parameters: TR = 2800 ms; TE = 408 ms; FOV = 256 mm × 256 mm × 192 mm; resolution = 1 mm isotropic; GRAPPA acceleration factor = 2; and acquisition time of 4 min and 30 s. Diffusion-weighted images (DWIs): Diffusion-weighted images (DWIs) are acquired with a twice-refocused spin-echo sequence, with 30 diffusion gradient directions for each of two b-values: 1000 and 2000 s/mm^2^, plus three images acquired with a b-value of 0. These parameters are optimized for the estimation of the diffusion kurtosis tensor and associated scalar metrics, as well as the traditional diffusion tensor. Other parameters are TR = 9100 ms; TE = 104 ms; voxel size = 2 mm isotropic; FOV = 192 mm × 192 mm; 66 axial slices; number of averages = 1; and acquisition time of 10 min and 2 s.

**Table A1:** Means of E-field amplitude (V/m) for different sexes. Data are represented as male (female).

Age (Years)	F3-F4	F3-P3	P3-P4	Fp1-P4
18–20	0.630 (0.639)	0.710 (0.675)	0.590 (0.588)	0.653 (0.607)
21–30	0.611 (0.596)	0.675 (0.671)	0.587 (0.582)	0.621 (0.610)
31–40	0.570 (0.571)	0.599 (0.664) **	0.550 (0.577)	0.565 (0.614) **
41–50	0.553 (0.553)	0.575 (0.636) ***	0.532 (0.550)	0.542 (0.589) **
51–60	0.551 (0.520)	0.580 (0.602)	0.518 (0.535)	0.538 (0.554)
61–70	0.543 (0.529)	0.574 (0.607)	0.516 (0.544)	0.533 (0.579) **
71–80	0.520 (0.516)	0.555 (0.588)	0.497 (0.534) **	0.518 (0.565) **
81–88	0.603 (0.543)	0.625 (0.633)	0.552 (0.560)	0.589 (0.579)

**: *p* < 0.05; ***: *p* < 0.001.

**Table A2:** Means of focality (cm3) for different sexes. Data are represented as male (female).

Age (Years)	F3-F4	F3-P3	P3-P4	Fp1-P4
18–20	7093.627 (7799.388)	8112.669 (8994.119)	15,074.122 (13,346.022)	7432.177 (7078.626)
21–30	6307.498 (6477.617)	7905.436 (7981.395)	12,606.733 (14,776.818) **	7428.422 (6767.201)
31–40	6737.324 (6891.327)	8531.659 (7292.458) **	12,406.133 (13,974.758) **	7012.688 (6430.987) **
41–50	6267.261 (6000.679)	8220.783 (6717.638) ***	12,371.470 (13,165.953)	6823.989 (6217.212) **
51–60	6279.537 (5997.522)	7812.807 (6797.435) **	11,852.961 (12,281.317)	6530.629 (6282.867)
61–70	5800.709 (5739.736)	6987.422 (6641.700)	10,638.890 (11,326.190)	6223.979 (5433.416) **
71–80	5876.427 (5500.898)	6338.130 (5683.273) **	10,270.464 (9783.348)	6057.178 (5075.829) ***
81–88	5103.779 (5294.637)	6188.709 (5085.589) **	8566.855 (8907.962)	5835.409 (5440.269)

**: *p* < 0.05; ***: *p* < 0.001.
